# Amputations

**Published:** 1862-04

**Authors:** 


### AMPUTATIONS.

The Sanitary Committee on the Treatment of Amputation, consisting of George Hayward, M. D., S. D. Townsend, M. D., John Ware, M. D., J. M. Warren, M. D., S. Carbot, Jr., M. D., and R. M. Hodges, M. D., report as follows:

The following paper, prepared by Dr. D. D. Slade, of Boston, is recommended for publication to the U. S. Sanitary Commission, by the Medical Commission of the State of Massachusetts.

*Necessity of Amputation.*—1. Cases where a limb is nearly, or completely carried away, leaving a ragged stump, with laceration of the soft parts, and projection of the bone.

2. Cases in which the soft parts of a limb are extensively lacerated or contused, the principal arterial and nervous trunks destroyed, and the bone denuded or fractured.

3. Cases in which a similar condition exists, without either fracture or denudation of the bone.

4. Cases of compound and comminuted fracture, particularly those involving joints.

5. Gun-shot wounds in which the ball does not actually penetrate the joint, but in which the bone being struck above or below, the fracture extends into the joint.

6. Gun-shot wounds between the phalanges of the fingers or toes, do not necessitate amputation.

7. Gun-shot wounds penetrating the wrist, unless great laceration has occurred, do not necessarily demand amputation.

8. In gun-shot injuries of the shoulder and elbow joints, provided the main blood-vessels and nerves are not injured, excision may be practiced with a fair prospect of success.

9. Compound fractures of the middle, and lower part of the thigh, occasioned by gun-shot, require amputation. As regards similar injuries in the upper two-thirds of the thigh, the mortality following amputations has been so very great that army surgeons have generally abandoned the operation.

Dr. McLeod, after a careful inquiry into this point, says: "Under circumstances of war, similar to those which occurred in the east, we ought to try to save compound comminuted fractures of the thigh, when situated in the upper third; But immediate amputation should be had recourse to in the case of a like accident occurring in the middle, and lower third."

Such cases must be left to the judgment of the Surgeon.

10. Gun-shot wounds of the knee-joint demand amputation. The operation of excision, in the very few cases in which it has been practised by army surgeons, has not been attended by favorable results. This want of success is not, however, to condemn, except upon the field of battle, an operation which has been so successfully performed in cases of disease.

11. Gun-shot fractures in the middle of the leg do not necessitate amputation, unless the arteries are destroyed, or the injuries involve the neighboring joints.

12. Gun-shot injuries of the ankle do not necessarily require amputation. If the posterior tibial artery and nerve have escaped injury, and if the bones be not too extensively comminuted, attempts may be made to save the limb.

13. Great care should be exercised, before proceeding to amputation, to ascertain whether a patient may be otherwise mortally wounded.

*The Time for Operating.*—In army-practice, on the field, amputation when necessary, ought to be primary. Patients, in most cases, cannot bear removal from the field without increased danger, neither can they have afterwards the hygienic attentions which secondary amputations must neces sarily require. Therefore:

1. Amputate with as little delay as possible, after the receipt of the injury, in those cases where there is intense suffering from the presence in the wound of spicula of bone, or other foreign bodies, which the fingers or forceps cannot reach.

2. In those cases where a limb is nearly torn off, and a dangerous hemorrhage is occurring, which cannot be arrested.

3. In those cases where it is *clearly* seen that the patient is not suffering from immediate collapse, or great nervous depression, a condition which will probably come on if there is any considerably delay. If the shock or collapse is extreme, the operation must be postponed, until, by appropriate measures, re-action is sufficiently established.

4. In certain cases, where the collapse is not extreme, the use of Sulphuric Ether, as an anæsthetic agent, often has the effect of bringing about moderate re-action. Such cases would formerly have required delay.

5. In army-practice, attempts to save a limb which might be perfectly successful in civil life, cannot be made. Especially is this the case in compound gun-shot fractures of the thigh, bullet wounds of the knee-joint and similar injuries of the leg, in which, at first sight, amputation may not seem necessary. Under such circumstances, attempts to preserve a limb, will be followed by extreme local and constitutional disturbance. Conservative surgery is here an error; in order to save life, the limb must be sacrificed. Moreover, the suppuration and sloughing, attendant upon mutilated limbs, soon render the atmosphere of over-crowded hospitals or barracks perfectly untenable; a fact entitled to a certain amount of weight, in cases where the propriety of primary amputation is at all questioned.

*The point of selection.*—Modern surgery has abundantly shown, that, as a general rule, the risk is greater in proportion as the size of the part which is amputated increases, and as the line of amputation approaches the trunk; in fact, the nearer to the trunk, the greater the danger. Therefore:

1. As a general rule, other things being equal, save as much of the limb as possible.

2. When time is of consequence, disarticulation of a phalanx is sometimes preferable to the division of the bone in its continuity. Disarticulation of the toes is always preferable, except, in some cases, the first phalanx of the great toe may be divided through its middle portion.

3. However extensive may be an injury to the hand, endeavors should be made to save a portion of it, if it be only one or two fingers. Especially should an attempt be made to preserve the thumb, and even in the very worst looking cases, such is the great reparative power of nature in these parts, that the surgeon may generally accomplish much in this respect.

4. Where time is of consequence, and even in most cases, disarticulation, at the wrist-joint is preferable to an attempt to save a few of the carpal bones.

5. In gun-shot injuries of the foot, attempts may be made to save a portion of the member by either of the methods recommended by Hey, Chopart, Pirogoff, or Syme. In place of Hey's operation, the disarticulation of the metatarsal bones from the tarsus being often troublesome, it is better to saw through the metatarsus just in front of the tarsal articulations. Should disarticulation at the ankle-joint be practiced, the removal of the malleoli must not be forgotten.

6. Other things being equal, it is best to save as much of the leg as possible, not exceeding three-fourths, in order for the better adaptation of an artificial limb.

7. In the rare cases which admit of its adoption, excision of the head of the femur is to be performed in preference to disarticulation, as being the least likely to lead to a fatal issue. When it is determined to perform amputation, it should, if possible, be made through the trochanters of the femur, rather than at the hip-joint.

8. In selecting the point for amputation, it must be remembered that, in gun shot wounds, the injuries are often far more extensive than they at first sight appear. Care therefore should be taken that the anxiety to preserve as much of the limb as possible, does not influence the Surgeon's better judgment, to the detriment, and perhaps even to the loss, of his patient, from subsequent sloughing and gangrene.

*Hints for after-treatment.*—1. When a wound is extensive, as in cases of amputation, it is far preferable to leave the wound open, with a piece of wet lint, or a thin compress, interposed between the lips, for two or three hours, until the surface has become glazed. In this way, as re-action comes on, hemorrhage may be often avoided, or if it does occur, is easily controlled without the disturbance of the dressings.

There need be no fear as regards the number of the ligatures applied. It is better to employ too many than too few, at the time of operation.

2. The dressings of a stump should be as simple and as little cumbersome as the case will in any way admit of. A narrow strip of water-dressing should be laid along the edge of the incision, over the strips of adhesive plaster, and the part should be so arranged that one end of the incision may be most dependent, in order to facilitate the escape of all discharges. An outlet for this purpose should never be neglected.

3. The position of the stump is of the utmost importance. By proper attention to this point, the edges and surfaces of the incision may be brought into contact, and the patient is spared the pain and uneasiness which, under other circumstances, the tension and pressure, necessary to bring the parts together, must invariably produce.

4. If the dressings are properly applied, as a general rule, these need not be changed for several days after amputation. Much mischief is undoubtedly done by a too hasty removal of the first dressings.

5. After removal of the first dressings, if union has not taken place by adhesive inflammation, and suppuration has commenced, with much heat and tenderness about the part, a poultice may be advantageously substituted for the water-dressing.

6. In all cases where there is much suppuration, and tendency to bagging of matter, the parts must be well supported by bandages.

7. Although complete primary union is desirable, the Surgeon should not be over-anxious to bring about this result.

8. Of course, in cases, where, after amputation of the patient to any considerable distance is contemplated, or likely to occur, the dressings must be so arranged, that any such removal will not disturb the parts, and thus interfere with the safety, or speedy recovery of the individual.

